# Reciprocal Regulation of Reactive Oxygen Species and Phospho-CREB Regulates Voltage Gated Calcium Channel Expression during *Mycobacterium tuberculosis* Infection

**DOI:** 10.1371/journal.pone.0096427

**Published:** 2014-05-05

**Authors:** Arti Selvakumar, Cecil Antony, Jhalak Singhal, Brijendra K. Tiwari, Yogendra Singh, Krishnamurthy Natarajan

**Affiliations:** 1 From the Infectious Disease Immunology Lab, Dr. B. R. Ambedkar Centre for Biomedical Research, University of Delhi, Delhi, India; 2 CSIR-Institute of Genomics and Integrative Biology, Delhi, India; University of KwaZulu-Natal, South Africa

## Abstract

Our previous work has demonstrated the roles played by L-type Voltage Gated Calcium Channels (VGCC) in regulating *Mycobacterium tuberculosis* (*M. tb*) survival and pathogenesis. Here we decipher mechanisms and pathways engaged by the pathogen to regulate VGCC expression in macrophages. We show that *M. tb* and its antigen Rv3416 use phospho-CREB (pCREB), Reactive Oxygen Species (ROS), Protein Kinase C (PKC) and Mitogen Activated Protein Kinase (MAPK) to modulate VGCC expression in macrophages. siRNA mediated knockdown of MyD88, IRAK1, IRAK2 or TRAF6 significantly inhibited antigen mediated VGCC expression. Inhibiting Protein Kinase C (PKC) or MEK-ERK1/2 further increased VGCC expression. Interestingly, inhibiting intracellular calcium release upregulated antigen mediated VGCC expression, while inhibiting extracellular calcium influx had no significant effect. siRNA mediated knockdown of transcription factors c-Jun, SOX5 and CREB significantly inhibited Rv3416 mediated VGCC expression. A dynamic reciprocal cross-regulation between ROS and pCREB was observed that in turn governed VGCC expression with ROS playing a limiting role in the process. Further dissection of the mechanisms such as the interplay between ROS and pCREB would improve our understanding of the regulation of VGCC expression during *M. tb* infection.

## Introduction

Tuberculosis caused by *Mycobacterium tuberculosis* (*M. tb*) results in high mortality and morbidity of individuals in the world. In 2012 an estimated 8.6 million new cases of TB (13% co-infected with HIV) was observed and 1.3 million people died from TB, including almost one million deaths among HIV-negative individuals [1].

Delineating the intricate network during host-pathogen interactions at the molecular and cellular levels is key to the development of vaccines and therapeutics. One such molecule that plays a central role in the above interactions is calcium. A number of reports have highlighted the importance of calcium in the pathogenesis of *M. tb* and the various ways by which the pathogen counteracts its protective effects [2].

Calcium fluxes in response to various stimuli govern the selective activation and inactivation of transcription factors resulting in altered genotypic and phenotypic outcomes [3]. A typical calcium response consists of two phases [4]. The first phase is the depletion of intracellular stores from the endoplasmic reticulum followed by the capacitative phase that is mediated by the activation of store operated calcium channels, thus leading to a sustained increase in intracellular calcium concentrations [5]. This second phase of calcium influx is either via calcium release calcium activated (CRAC) channels or via Voltage Gated Calcium Channels (VGCC) or both [6]. Although extensively studied in physiological states and disorders [7]–[8], the role of VGCC in infections is now beginning to be appreciated [9], [10], [11].

We previously reported the roles played by VGCC during infection by *M. tb* [12]. Briefly, inhibiting L-type or R-type VGCC in DCs and PBMCs increased calcium influx upon infection. This resulted in enhanced expression of pro-inflammatory genes that play critical roles in protective immunity. Importantly, knockdown of L-type or R-type VGCC resulted in reduced intracellular burden of virulent *M. tb*. Further, blocking L-type or R-type VGCC in DCs, activated T cells that mediated killing of *M. tb* inside macrophages. Significantly, PBMCs of patients with active TB disease expressed high levels of VGCC that were attenuated following chemotherapy. Finally, blocking L-type and R-type VGCC in mice infected with virulent *M. tb* resulted in reduced bacterial burden. Similar results were later obtained by Ramakrishnan and co-workers [13], whereby inhibiting L-type VGCC resulted in attenuated intracellular mycobacterial burden. Further, Gupta et al have recently demonstrated the ability of the calcium channel blocker verapamil to accelerate the clearance of *M. tb* infection in mice [14].

Therefore, given the role of calcium in regulating protective responses to *M. tb* and the roles played by VGCC therein, in this report, we elucidated the mechanisms by which *M. tb* and its antigens regulate the expression of VGCC on macrophages. Our results indicate that *M. tb* and its antigens regulate L-type VGCC expression in a TLR dependent manner and we identify both positive and negative mediators of VGCC expression. Further delineation of the roles of these mediators, especially the contrasting roles of intracellular and extracellular calcium release and the interplay between ROS and pCREB would increase our understanding on the roles played of VGCC in regulating calcium homeostasis and its regulation by *M. tb*.

## Materials and Methods

### Cell Culture and Differentiation

Monocyte macrophage cell line THP1 was used throughout the study. THP-1 was a kind gift from Dr. Pawan Sharma from International Centre for Genetic Engineering and Biotechnology, New Delhi [15]. Cells were maintained in RPMI1640 medium supplemented with 10% FBS and 2 mmol/L L-glutamine. THP1 cells were differentiated into macrophages by incubation with 50 ng/ml of PMA for 16h. The following biopharmacological inhibitors were used against various molecules. PKC, calphostin C (0.1 µM); PI3-K, Wortmanin (10 nM); Calmodulin kinase II, KN62 (10 µM); MAPK-ERK U0126 (10 µM); IP_3_R, 3,4,5-trimethoxybenzoic acid 8-(diethylamino)octyl ester (TMB-8) (100 µM); calcium influx, EGTA (3 mM), ROS, Diphenyleneiodonium (DPI) (10 µM); ROS, N-Acetyl Cysteine (NAC) (50 mM). Additionally, where mentioned, cells were stimulated with 50 µM H_2_O_2_ for indicated times_._ Unless mentioned otherwise, cells were incubated with the above reagents for 1h prior to stimulation with Rv3416.

### Materials

Antibodies to pCREB-1, SOX5, NF-κBp65, c-Jun, specific and control siRNAs to various genes and Luminol kits for chemiluminescence detection were purchased from Santa-Cruz Biotechnologies (Santa Cruz, CA). Recombinant *M. tb* antigen Rv3416 was expressed and purified as described before [16], [17]. U0126, KN62, TMB8, EGTA, Calphostin C, DPI and Wortmanin were purchased from Sigma Chemical Co. (St. Louis MA).

### Flow Cytometry

Antibody to L-type Ca^2+^ α1C (Santa Cruz Biotechnologies catalog # sc-25686) was biotinylated using NHS biotin as per standard protocols. Cells were first incubated with Fc-block (BD Biosciences) followed by incubation with the above antibody at 1 µg/10^6^ cells at 4°C for 30 min. Cells were washed and counter-stained with either streptavidin-PE or streptavidin-FITC and acquired on FACS Calibur (Beckton & Dickinson) and the data were analyzed using CellQuest Pro software. No gates were applied for analyses.

### Transfection of THP1 cells with siRNA and stimulations

1×10^6^/ml PMA differentiated THP1 cells were transfected with 60pmoles of siRNA against various genes for 36h using the Hiperfect transfection reagent (Qiagen) in OPTIMEM medium (Invitrogen) as described earlier [17]. Subsequently, cells were stimulated with Rv3416 for 72h and analyzed by FACS. Figure S1 shows the knockdown efficiency of various genes used in the study. ROS levels were monitored as described earlier [17]. Briefly, 30min prior to the incubation period, cells were loaded with 10 µM DCFH-DA. At the end of the incubation period, cells were thoroughly washed with culture medium. Cells were washed once again with culture medium and immediately analyzed for ROS levels by flow cytometry.

### Western blotting for signaling molecules

At the end of incubation, cells were chilled on ice, washed once with ice cold PBS, and lysed in buffer containing 10 mM HEPES (pH 7.9), 10 mM KCl, 0.1 mM EDTA, 0.1 M EGTA, 0.5% Nonidet P-40, and 2 µg/ml each aprotinin, leupeptin and pepstatin. The suspension was centrifuged at 13,000 g for 5min at 4°C. The pellet was solubilized for 30min with intermittent vortexing with buffer containing 20 mM HEPES (pH 7.9), 0.4 M NaCl, 1 mM EDTA, 1 mM EGTA and 2 µg/ml each aprotinin, leupeptin and pepstatin. The suspension was then centrifuged at 13,000 g for 15min at 4°C. The supernatant was designated as the nuclear extract. 25 µg of protein was resolved on 10% SDS-PAGE and subsequently transferred onto nitrocellulose membrane (Hybond C pure, Amersham Biosciences, Arlington Heights, IL). The blots were then probed with Abs to various molecules, followed by HRP-labeled secondary Abs. The blots were later developed by chemiluminescence using the luminol reagent.

### Confocal microscopy

At the end of incubation cells were fixed with acetone:methanol::1∶1 for 20 min in 4°C. Cells were washed twice and incubated with antibodies against L-type Ca^2+^ α1C for 2h followed by addition of anti-rabbit FITC tagged Alexa flour 488 for 1.5h. Cells were again washed and mounted with anti-fade containing DAPI. Confocal imaging was performed on Nikon C2 laser scan confocal microscope (Nikon, Japan) with 60X objective magnification, numerical aperture 1.4, refractive index 1.5, Plan Apo optics equipped with Argon laser, using excitation and emission wavelength of 488 nm, respectively. Data were analyzed using the NIS Elements AR software.

## Results

### *M. tb* antigen Rv3416 induces L-type VGCC expression on macrophages

**Figure 1:**
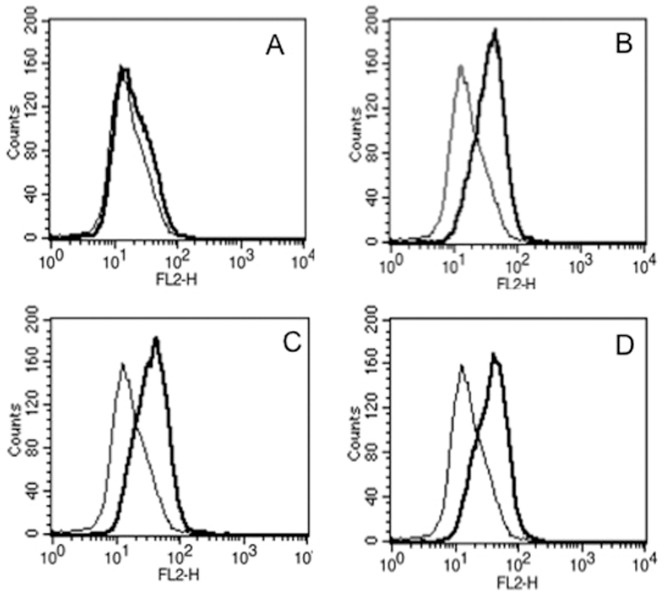
Rv3416 induces the upregulation of L-type VGCC on macrophages. THP1 cells were stimulated overnight with 50/ml PMA followed by Rv3416 stimulation for 72h. L-type VGCC expression was monitored by flow cytometry. Bold lines represent stimulations with Rv3416 while thin lines represent unstimulated controls. Panels A-D represent stimulation with 15 µg/ml, 30 µg/ml, 45 µg/ml and 60 µg/ml of Rv3416, respectively. Data from one of four independent experiments are shown.

To begin with we examined the ability of Rv3416 to modulate VGCC expression on macrophages. Rv3416 is an antigen we identified previously that is expressed in macrophages as a function of infection and time [16]. Functional characterization indicated that Rv3416 mediates immune suppression by preventing maturation of DCs, downmodulates secretion of IL-12p40 together with downmodulation of surface HLA, IL-12 receptor and IFN-γ receptor expression on macrophages. Further it also inhibits pro-inflammatory T cell responses to *M. tb* [16]. Priming DCs and macrophages with Rv3416 increased *M. tb* burden. Since VGCC played a major role in immune evasion to *M. tb*, we therefore, investigated the role of Rv3416 in mediating VGCC expression in macrophages. As shown in Figure 1, stimulation of macrophages increased VGCC expression at all doses, with maximal expression at 72h post-stimulation with 30 µg/ml, indicating that Rv3416 played a positive role in mediating VGCC expression. No significant expression of VGCC over and above unstimulated controls was observed at earlier time points (data not shown).

### Rv3416 induces VGCC expression in a TLR dependent manner

**Figure 2:**
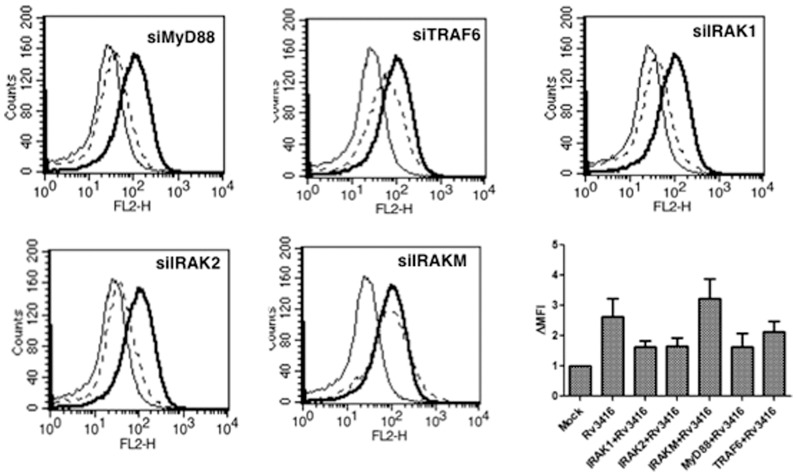
Rv3416 upregulates VGCC in a TLR dependent pathway. PMA stimulated THP1 cells were transfected with siRNA against MyD88, TRAF6, IRAK1, IRAK2 or IRAKM for 36h, followed by stimulation with 30 µg/ml of Rv3416 for 72h. L-type VGCC levels were monitored using flow cytometry. Bold lines represent cells transfected with control siRNA followed by stimulations with Rv3416, while dotted lines represent cells transfected with specific siRNA to indicated molecules followed by stimulations with Rv3416. Thin lines represent unstimulated cells transfected with control siRNA. One of three independent experiments is shown. Bar chart shows the Fold increase in Mean Fluorescence Intensity (ΔMFI) in different groups over and above unstimulated control (represented as MOCK). Bars represent mean ± S.D. of three independent experiments.

As Toll-like receptor (TLR) signaling pathways play major roles in regulating immunity to pathogens, we next investigated whether, Rv3416 mediated VGCC expression involved TLR induced pathways. To that end using specific siRNA, we knockdown MyD88, IRAK1, IRAK2, IRAKM or TRAF6 prior to stimulation with Rv3416. As shown in Figure 2, knockdown of MyD88, IRAK1, IRAK2, or TRAF6 but not IRAKM significantly inhibited Rv3416 mediated VGCC expression. This indicated that Rv3416 induced expression of VGCC indeed involved the TLR pathway. Since IRAKM is a known negative regulator of MyD88 dependent TLR signaling [18] the above results are therefore in concurrence with its reported role. No significant changes in VGCC expression was observed upon stimulation of cells with known ligands to TLR2, TLR4, TLR7 or TLR9 (data not shown).

### Protein Kinase C and MAPK-ERK negatively regulate VGCC expression by *M. tb* antigens

**Figure 3:**
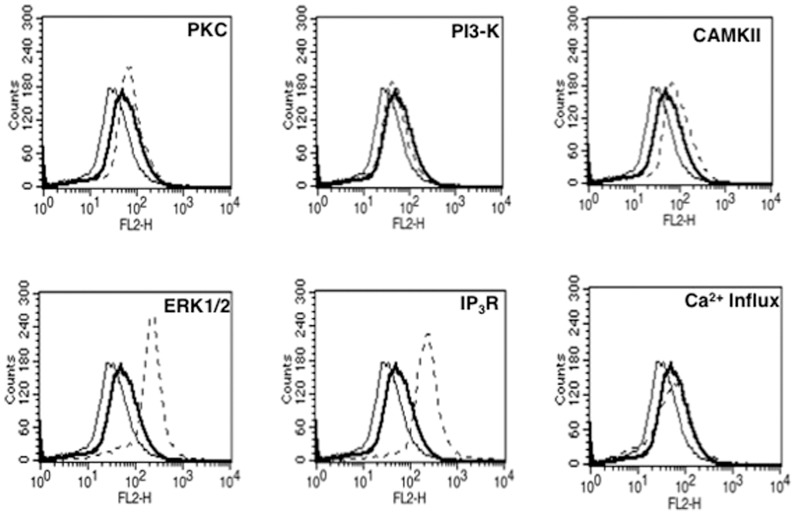
PKC, ERK/MAPK, CAMKII and intracellular calcium release negatively regulate Rv3416 mediated VGCC expression. PMA stimulated THP1 cells were incubated with inhibitors to indicated molecules (*see Experimental Procedures*) for 1h prior to stimulation with Rv3416. L-type VGCC levels were monitored by FACS 72h post stimulation. Bold lines represent cells stimulated with 30 µg/ml of Rv3416, while dotted lines represent cells incubated with inhibitors to indicated molecules followed by stimulations with Rv3416. Thin lines represent unstimulated cells. One of four independent experiments is shown.

In order to further dissect the pathways that lead to Rv3416 mediated VGCC expression we inhibited key signaling intermediates using specific bio-pharmacological inhibitors and monitored the expression levels of VGCC following specific stimulations. As shown in Figure 3, inhibiting either PKCα, MAPK-ERK1/2, calmodulin kinase II (CAMKII) or Inositol 1,4,5 triphosphate (IP_3_) receptor resulted in a further increase in the levels of L-type VGCC. This indicated that these intermediates play a negative role in expression of L-type VGCC by Rv3416. On the other hand, inhibiting either phosphoinositide 3-kinase (PI3-K) or external calcium influx had no significant effect. It is interesting to note a negative regulation of a calcium channel by an internal store operative pathway, with no significant effect by the extracellular calcium influx. This also indicates that not only is calcium a key regulator of VGCC expression, but its routing from different channels is also a critical component mediating regulation.

In order to investigate whether, the results obtained with Rv3416 are in concurrence with live infections we performed key experiments with live mycobacteria. To that end, macrophages were infected with live *M. tb* and VGCC expression levels were monitored. As shown in Figure S2, *M. tb* infection also resulted in a significant upregulation of VGCC expression, indicating that live mycobacteria also upregulates VGCC expression on macrophages that was further enhanced following inhibiting PKC or MAPK-ERK. We also monitored the levels of VGCC by confocal microscopy. As shown in Figure S3, stimulation with Rv3416 increased VGCC expression over and above those in unstimulated controls. The levels were further enhanced following inhibiting either PKC or MAPK-ERK.

### CREB, c-jun and SOX5 positively regulate VGCC expression by Rv3416

**Figure 4:**
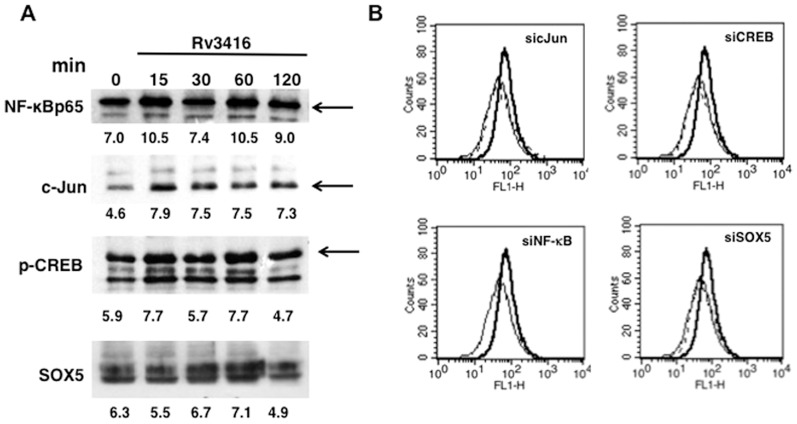
CREB, c-Jun and SOX5 positively regulate Rv3416 mediated VGCC expression on macrophages. For Panel A, PMA stimulated THP1 cells were stimulated with 30 µg/ml of Rv3416 for indicated times and the levels of indicated transcription factors were monitored in nuclear extracts by western blots. Numbers below the blots indicate relative intensities of the bands. Arrow indicates the specific band based on molecular weight markers. Data from one of two experiments are shown. For Panel B, PMA stimulated THP1 cells were transfected with siRNA against c-Jun (sicJun), CREB (siCREB), NF-KBp65 (siNF-κB) and SOX5 (siSOX5) for 36h, followed by stimulation with 30 µg/ml of Rv3416 for 72h. VGCC levels were monitored using flow cytometry. Bold lines represent cells transfected with control siRNA followed by stimulations with Rv3416, while dotted lines represent cells transfected with specific siRNA to indicated transcription factors followed by stimulations with Rv3416. Thin lines represent unstimulated cells transfected with control siRNA. One of two independent experiments is shown.

In order to identify transcription factors that play a role in regulating Rv3416 mediated VGCC expression, we monitored the nuclear levels of key transcription factors that regulate inflammatory responses to *M. tb* and its antigens. As shown in Figure 4A, stimulation with Rv3416 significantly activated NF-κBp65, phospho^Ser133^-CREB (pCREB), c-Jun and SOX5. Next, in order to investigate whether the activated transcription factors would indeed regulate VGCC expression, we individually knockdown these transcription factors using siRNA and monitored Rv3416 mediated VGCC expression. As shown in Figure 4B, knockdown of either c-Jun, SOX5 or CREB completely inhibited Rv3416 induced VGCC expression indicating a requirement for all the three transcription factors in VGCC expression. Interestingly, no effect of knockdown of NF-κBp65 on VGCC expression was observed, indicating no significant role for this transcription factor in VGCC expression.

### Reactive Oxygen Species (ROS) regulate Rv3416 induced VGCC expression

**Figure 5:**
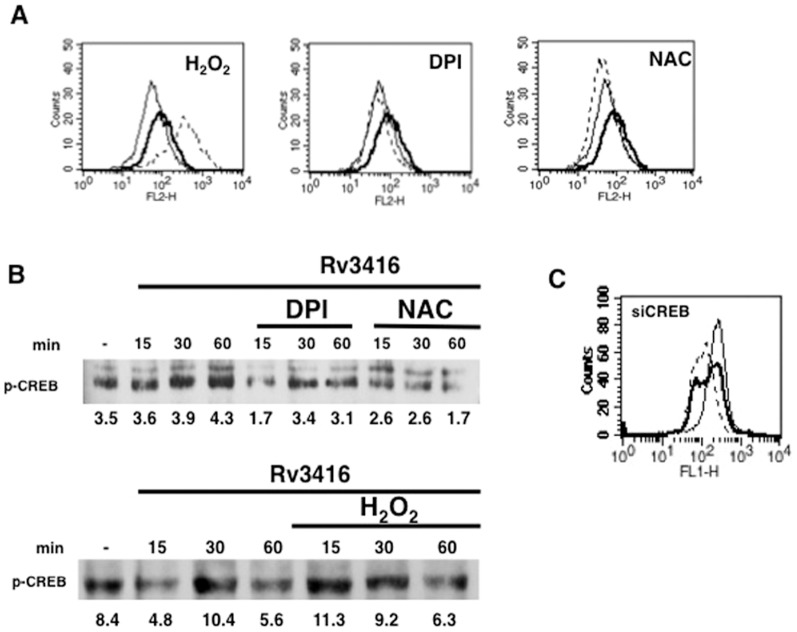
Reciprocal regulation between ROS and pCREB regulates VGCC expression. For Panel A, PMA stimulated THP1 cells were incubated with indicated regents (*see Experimental Procedures*) for 1h followed by stimulation with 30 µg/ml of Rv3416. L-type VGCC levels were monitored after 72h by FACS. Bold lines represent cells stimulated with Rv3416, while dotted lines represent cells incubated with indicated reagents followed by stimulations with Rv3416. Thin lines represent unstimulated cells. For Panel B, PMA stimulated THP1 cells were stimulated with 30 µg/ml of Rv3416 with or without indicated reagents for indicated times and the nuclear levels of pCREB were monitored by western blot. Numbers below the blots indicate relative intensities of the bands. Panel C shows ROS levels in PMA stimulated THP1 cells transfected with siRNA to CREB (siCREB) followed by stimulation with Rv3416 for 2h. Bold lines represent cells transfected with control siRNA followed by stimulation with Rv3416, while dotted lines represent cells transfected with siRNA to CREB (siCREB) followed by stimulations with Rv3416. Thin lines represent unstimulated cells transfected with control siRNA. Data from one of three experiments are shown.

Reciprocal regulation between ROS and calcium homeostasis has often been reported [19]. In fact we reported the positive regulation of calcium influx by ROS following activation of dendritic cells by Rv3874 (CFP10) [20], an *M. tb* specific antigen that is co-expressed along with Rv3875 (ESAT6). Rv3874 plays a determinant role in the survival of mycobacteria inside DCs, such that increasing oxidative burst in DCs reduced bacterial burden. Further, we recently showed that Rv3416 downmodulates ROS in dendritic cells [17]. Therefore, we next investigated the role of ROS in regulating Rv3416 mediated VGCC expression. As shown in Figure 5A, while increasing intracellular ROS levels with H_2_O_2_ further increased VGCC expression, inhibiting intracellular ROS prevented Rv3416 mediated VGCC expression. This indicated that ROS played a positive role in regulating Rv3416 mediated VGCC expression. It is interesting to note that while ROS played a protective role in DCs, it seems to promote immune evasive responses in macrophages at least in the context of VGCC. It has often been reported that *M. tb* and its antigens induce both similar and contrasting responses in DCs and macrophages [21].

In order to investigate the contrasting results obtained above, wherein stimulation of macrophages by Rv3416 downmodulated ROS levels yet upregulated VGCC; and at the same time supplementing ROS upregulated VGCC, we investigated the reciprocal regulation of ROS and pCREB. As shown in Figure 5B, ROS positively regulated pCREB activation since inhibiting ROS by NAC or DPI reduced Rv3416 mediated pCREB activation; and conversely, supplementing ROS with H_2_O_2_ further enhanced Rv3416 mediated pCREB activation with advanced kinetics. We also investigated whether pCREB could reciprocally regulate ROS generation during Rv3416 stimulation. To that end we knockdown CREB with specific siRNA followed by stimulation with Rv3416 and monitored ROS. As shown in Figure 5C, stimulation with Rv3416 downmodulated ROS and interestingly, knockdown of CREB further attenuated ROS levels over and above that mediated by Rv3416. This indicated that CREB also positively regulated ROS generation during Rv3416 stimulation. Put together, the data in Figure 5 point to an interesting cross-regulation of ROS and pCREB that governs VGCC expression by Rv3416 in macrophages. Specifically, both ROS and pCREB are required for VGCC expression by Rv3416. A downmodulation of ROS by Rv3416 is compensated with a concomitant increase in pCREB activation resulting in increased VGCC expression. Supplementing ROS increases pCREB activation thereby further enhancing VGCC expression by Rv3416. Knockdown of CREB further attenuates ROS over and above that mediated by Rv3416 resulting in attenuating VGCC expression. Reciprocally, inhibiting ROS also attenuates pCREB activation thereby resulting in reduced VGCC expression.

### PKC and MAPK-ERK regulate VGCC expression via ROS modulation

**Figure 6:**
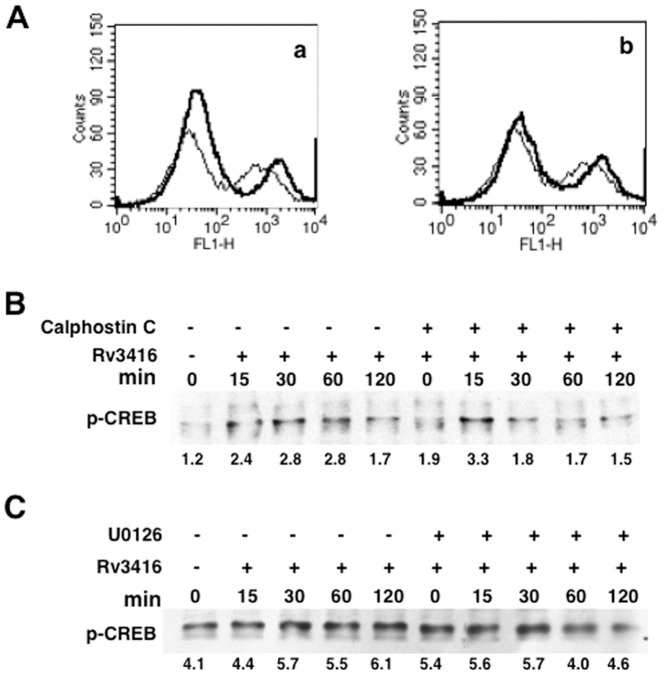
Inhibition of PKC or MAK-ERK enhances ROS generation in Rv3416 stimulated cells. For Panel A, PMA stimulated THP1 cells were incubated with an inhibitor to PKC (subpanel a) or MAPK-ERK (subpanel b) for 1h prior to stimulation with 30 µg/ml of Rv3416 for 2h and ROS levels were monitored by flow cytometry. Thin line represents unstimulated control while bold lines represent Rv3416 stimulated cells. For Panel B and C, PMA stimulated THP1 cells were incubated with inhibitor to PKC (Calphostin C, Panel B) or MAPK-ERK (U0126, Panel C) and stimulated with Rv3416 for indicated times. Nuclear extracts were probed for pCREB levels. Numbers below the blots indicate relative intensities of the bands. Data from one of three experiments is shown.

We extended these observations by investigating the role of PKC and MAPK in regulating ROS generation and pCREB activation. To that end, we monitored ROS levels following inhibition of PKC and MAPK-ERK during stimulations with Rv3416 and. As shown in Figure 6A, inhibiting either PKC (Figure 6A, panel a) or MAPK-ERK (Figure 6A, panel b) enhanced ROS levels. However, neither PKC nor MAPK/ERK significantly modulated pCREB activation (Figure 6B and C). A marginal advancement in the kinetics of activation was observed following PKC inhibition. This indicated that PKC and MAPK/ERK enhanced VGCC expression primarily by increasing ROS levels in Rv3416 stimulated macrophages with no significant modulation of pCREB activation. Interestingly, inhibiting IP_3_R or calmodulin Kinase II did not significantly modulate either ROS or pCREB activation, indicating the involvement of a different mechanism for upregulation of VGCC (data not shown).

### Specific genes in the calcium-calmodulin and cysteine protease pathways negatively regulate VGCC expression

**Figure 7:**
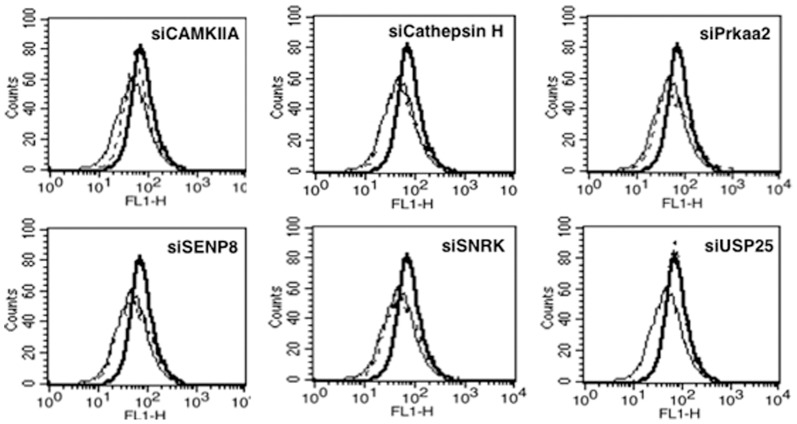
Specific genes in the calcium-calmodulin and cysteine protease pathways differentially regulate Rv3416 mediated VGCC expression. PMA stimulated THP1 cells were transfected with siRNA against CAMKIIA, Cathepsin H, Prkaa2, SENP8, SNRK or USP25 for 36h, followed by stimulation with 30 µg/ml of Rv3416 for 72h. VGCC levels were monitored using flow cytometry. Bold lines represent cells transfected with control siRNA followed by stimulations with Rv3416, while dotted lines represent cells transfected with specific siRNA to indicated molecules followed by stimulations with Rv3416. Thin lines represent unstimulated cells transfected with control siRNA. One of three independent experiments is shown.

Using siRNA libraries, we recently identified specific genes in the cysteine protease and calcium-calmodulin pathways that negatively regulated multiple responses from dendritic cells during *M. tb* infection [17]. Briefly, knockdown of these genes not only resulted in attenuating bacterial burden in DCs, but also induced pro-inflammatory cytokine and Th1 responses. Further, their knockdown also resulted in enhanced induction of autophagy that has now emerged as a potent anti-mycobacterial response [17]. Therefore, to investigate the role of these genes in mediating Rv3416 induced VGCC expression, we specifically knockdown these genes using siRNA followed by stimulation with Rv3416. As shown in Figure 7, knockdown of CAMKIIA, Cathepsin H, SNRK, SENP8 or Prakaa2 significantly inhibited Rv3416 mediated VGCC expression. No significant change was observed upon knockdown of USP25. The results indicate that these genes positively regulated VGCC expression. Further, these results also point towards another yet another mechanism of negative effects of these genes; wherein increased expression of VGCC expression could contribute towards attenuation of protective responses.

**Figure 8:**
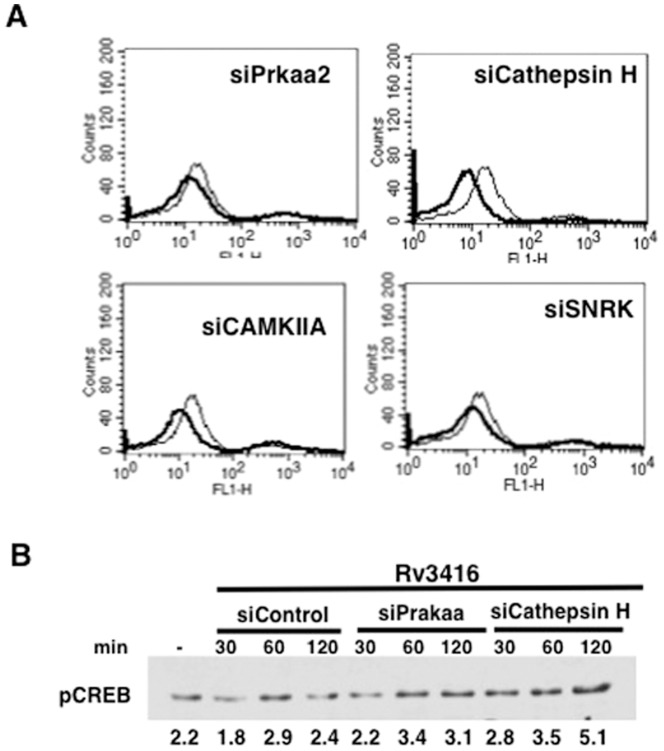
Genes in the calcium/calmodulin and cysteine protease pathways positively regulate ROS levels. For Panel A, PMA stimulated THP1 cells were transfected with siRNA against indicated genes and ROS levels were monitored by FACS 2h post stimulation with Rv3416. Bold lines represent cells transfected with siRNA against indicated genes while thin lines represent unstimulated cells transfected with control siRNA. Panel B shows pCREB levels in nuclear extracts of PMA stimulated THP1 cells transfected with siRNA against indicated genes followed by stimulation with Rv3416 for indicated times. One of two independent experiments is shown.

We next investigated the mechanisms by which these genes mediate VGCC expression. To that end we monitored both ROS generation and pCREB activation following knockdown of these genes followed by Rv3416 stimulation. As shown in Figure 8, knockdown of the genes differentially regulated ROS and pCREB activation. While knockdown of the genes further attenuated Rv3416 mediated ROS generation (Figure 8A). Interestingly, knockdown of either Prkaa2 or Cathepsin H increased pCREB activation (Figure 8B). No significant modulation in pCREB levels was observed upon knockdown of either CAMKIIA or SNRK (data not shown). These results establish a critical threshold of ROS below which no enhancement of VGCC expression is induced despite a concomitant increase in pCREB activation.

## Discussion

Amongst all the second messengers in the cell, calcium plays a central role in governing various responses against microbial infections. Calcium homeostasis in response to varied stimuli alters cellular dynamics to the extent of cell survival, proliferation or cell death. By virtue of regulating such diverse states, calcium concentration inside the cells is often modulated by invading pathogens as a primary immune evasive strategy [22]. Consequently, studying calcium regulation in response to infections has been a subject of intense research. During *M. tb* infection calcium regulates activation of calmodulin and sphingosine kinase affecting *M. tb* survival [23]. Calcinuerin regulates the expression of coronin-1 on phagosomes thus affecting phagosome maturation [24]. NF-AT, NF-κB, pCREB and c-Jun are differentially modulated as a result of calcium fluxes leading to differential cytokine expression that in turn regulates various aspects of immune responses [25]. Although, VGCC have been shown to play major roles in physiological responses, their roles in infectious diseases have recently assumed importance. For example, L-type VGCC has been demonstrated to play roles in regulating intracellular growth of *Legionella pneumophila* [10] and in induction of calcium in CD4^+^ T cells during *Leishmania* infection [11].

We reported the role of calcium in regulating immune responses to *M. tb* from dendritic cells (DCs) and macrophages [12]. Taking a cue from these results, we next explored calcium dynamics from VGCC. Our results showed that mycobacterial antigens and live infections upregulate the levels of VGCC on DCs and PBMCs [12]. Elevated VGCC prevents calcium influx in infected cells leading to increased survival of virulent mycobacteria. Inhibiting VGCC using siRNA enhanced intracellular calcium concentrations in cells and in infected mice that lead to decreased bacterial burden in vitro and in vivo [12]. This clearly showed that *M. tb* and its components increase the expression of VGCC as an immune evasive mechanism. Therefore, elucidation of mechanisms and pathways by which *M. tb* and its antigens modulate VGCC expression would further our understanding of calcium regulation during mycobacterial infection.

To this end we began investigating the role of a recently identified antigen in our lab that is expressed in infected macrophages as a function of time and plays an immune suppressive role in DCs and macrophages [16]. Our results showed that Rv3416 upregulated VGCC in a time and dose dependent manner. The upregulation involved TLR signaling intermediates, wherein knockdown of key modules significantly inhibited VGCC expression. Although TLR pathways are credited towards mediating protective responses, it has often been observed that microbes and/or their antigens modulate TLR pathways to their advantage by skewing putative protective responses towards suppressive responses. This is true for both bacteria and viruses. For example, *M. tb* 19 kDa lipoprotein utilizes the TLR2 pathway to subvert IFN-γ induced responses [26].

In order to further explore intermediates downstream of TLR signaling, we used bio-pharmacological inhibitors to key signaling second messengers in regulating VGCC expression. Our results identified both positive and negative regulators of VGCC expression. Inhibiting PKC, MAPK-ERK and IP_3_R inhibited Rv3416 induced VGCC expression indicating a negative regulatory effect of these molecules. Signaling downstream of TRAF6 from TLRs essentially diversifies into multiple modules [27]. One such pathway includes the activation of PKC and MAPK pathways. The fact that knockdown of TRAF6 prevented VGCC induction while inhibition of PKC or MAPK-ERK enhanced VGCC expression indicates a complex network of pathways regulating VGCC expression. TRAF6 appears to be a key positive regulator of VGCC expression since its knockdown inhibited VGCC expression despite a possible inhibition of PKC and/or MAPK-ERK. On the other hand, inhibition of PKC or MAPK could complement TRAF6 mediated induction of VGCC.

In the next set of experiments, we investigated the role of key transcription factors in regulating VGCC expression. We chose to focus on NF-κB, c-Jun and pCREB that are regulated by calcium and in turn regulate inflammatory responses [28], [29]. We also investigated the activation status of SOX5 that has a functional binding site in the mouse L-type VGCC promoter [30]. Stimulation with Rv3416 activated all the four transcription factors in a time dependent manner. Barring NF-κB the enhancement in activation also directly influenced VGCC expression since their knockdown prevented Rv3416 mediated VGCC expression.

We had earlier observed a cross-regulation between ROS, PKC and calcium in dendritic cells [20]. Briefly, increasing ROS in DCs prevented PKC activation but increased intracellular calcium levels. Reciprocally, inhibiting ROS increased activation of PKC and inhibited calcium influx in a dose dependent manner. In the next set of experiments we therefore examined the role for ROS in regulating antigen mediated VGCC expression. Our results showed that increasing ROS in cells enhanced VGCC expression, possibly by preventing PKC activation. Conversely, inhibiting ROS prevented Rv3416 mediated VGCC expression perhaps by enhancing PKC activation. Further, inhibiting either PKC or MAPK-ERK increased ROS levels thereby contributing towards enhanced VGCC expression.

In order to further dissect the pathways and identify key players during VGCC expression, we investigated the cross-regulation of ROS and pCREB by each other and its impact on VGCC expression. Our results point to a distinct reciprocal regulation of ROS and pCREB and a rate-limiting role for ROS during VGCC expression. The results identified a minimum threshold level of ROS required for VGCC expression that could not be compensated by increasing pCREB activation. These results point to a central and critical role for ROS during VGCC expression.

In order to further dissect the mechanism of VGCC upregulation, we investigated the role of genes in the calcium/calmodulin and cysteine protease pathways. Our recent study identified a set of genes from the above pathways that played a negative role during *M. tb* infection in DCs [17] **.** Knockdown of these genes resulted in mounting of pro-inflammatory responses against the pathogen. Our results suggest that some of these genes also contribute towards immune suppression by positively regulating VGCC upregulation. Knockdown of genes indeed prevented Rv3416 mediated VGCC induction and correlated with a reduction in ROS levels, despite a concomitant enhancement in pCREB activation. This once again reiterated a crucial role of ROS during VGCC expression.

Collectively, the results presented in the study have identified key mechanisms and intermediates that regulate the expression of VGCC in macrophages. A critical role for ROS and pCREB was observed. Cross-regulation of ROS and pCREB for VGCC expression acts in a synergistic and yet competitive manner, such that stimulations that enhance both, synergistically enhance VGCC expression. On the other hand, a stimulation that inhibits one but enhances the other also affects VGCC expression. These results indicate the involvement of multiple levels of regulation of VGCC by mycobacteria and its antigens.

## Supporting Information

Figure S1 **Knockdown efficiency of different genes.** PMA stimulated THP1 cells were transfected either with control siRNA or siRNA against indicated genes for 36h. Total RNA was extracted using Trizol and subjected to semi-quantitative RT-PCR. Upper lanes represent levels of specific genes following transfection with indicated siRNAs. Lower lanes depict corresponding levels of Actin.
(TIF)

Figure S2 ***M. tb*** **induces the upregulation of L-type VGCC on macrophages.** THP1 cells were infected with 2 MOI *M. tb* H37Rv for 72h. For some groups, cells were incubated with inhibitor to PKC (Calphostin C) or MAPK-ERK (U0126) for 1h prior to infection for 72h. At the end of the incubation period L-type VGCC expression was monitored by flow cytometry. Bars represent Mean Fluorescence Intensity.
(TIF)

Figure S3 **Confocal images of L-type VGCC following stimulations with Rv3416.** THP1 cells were stimulated overnight with 50 ng/ml PMA followed by Rv3416 stimulation with or without indicated reagents for 72h. L-type VGCC expression was monitored by confocal imaging as described in Experimental Procedures. A representative image of 10 fields is shown.
(TIF)
