# Supplementary figures and images for: Reciprocal Regulation of Reactive Oxygen Species and Phospho-CREB Regulates Voltage Gated Calcium Channel Expression during Mycobacterium tuberculosis Infection
Source: PLoS One. 2014 May 5;9(5):e96427. doi: 10.1371/journal.pone.0096427 (PMC4010530; doi:10.1371/journal.pone.0096427)

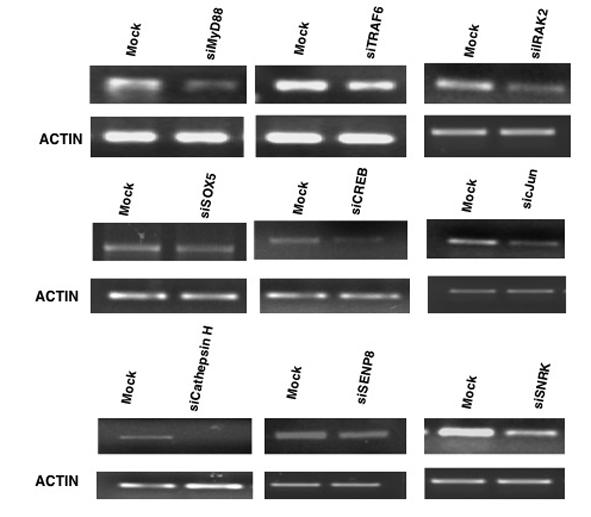

Supplement: Figure S1 — Knockdown efficiency of different genes. PMA stimulated THP1 cells were transfected either with control siRNA or siRNA against indicated genes for 36h. Total RNA was extracted using Trizol and subjected to semi-quantitative RT-PCR. Upper lanes represent levels of specific genes following transfection with indicated siRNAs. Lower lanes depict corresponding levels of Actin. (TIF) [file pone.0096427.s001.tif]

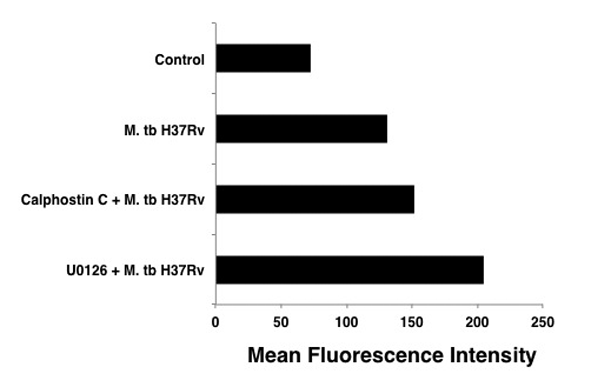

Supplement: Figure S2 — M. tb induces the upregulation of L-type VGCC on macrophages. THP1 cells were infected with 2 MOI M. tb H37Rv for 72h. For some groups, cells were incubated with inhibitor to PKC (Calphostin C) or MAPK-ERK (U0126) for 1h prior to infection for 72h. At the end of the incubation period L-type VGCC expression was monitored by flow cytometry. Bars represent Mean Fluorescence Intensity. (TIF) [file pone.0096427.s002.tif]

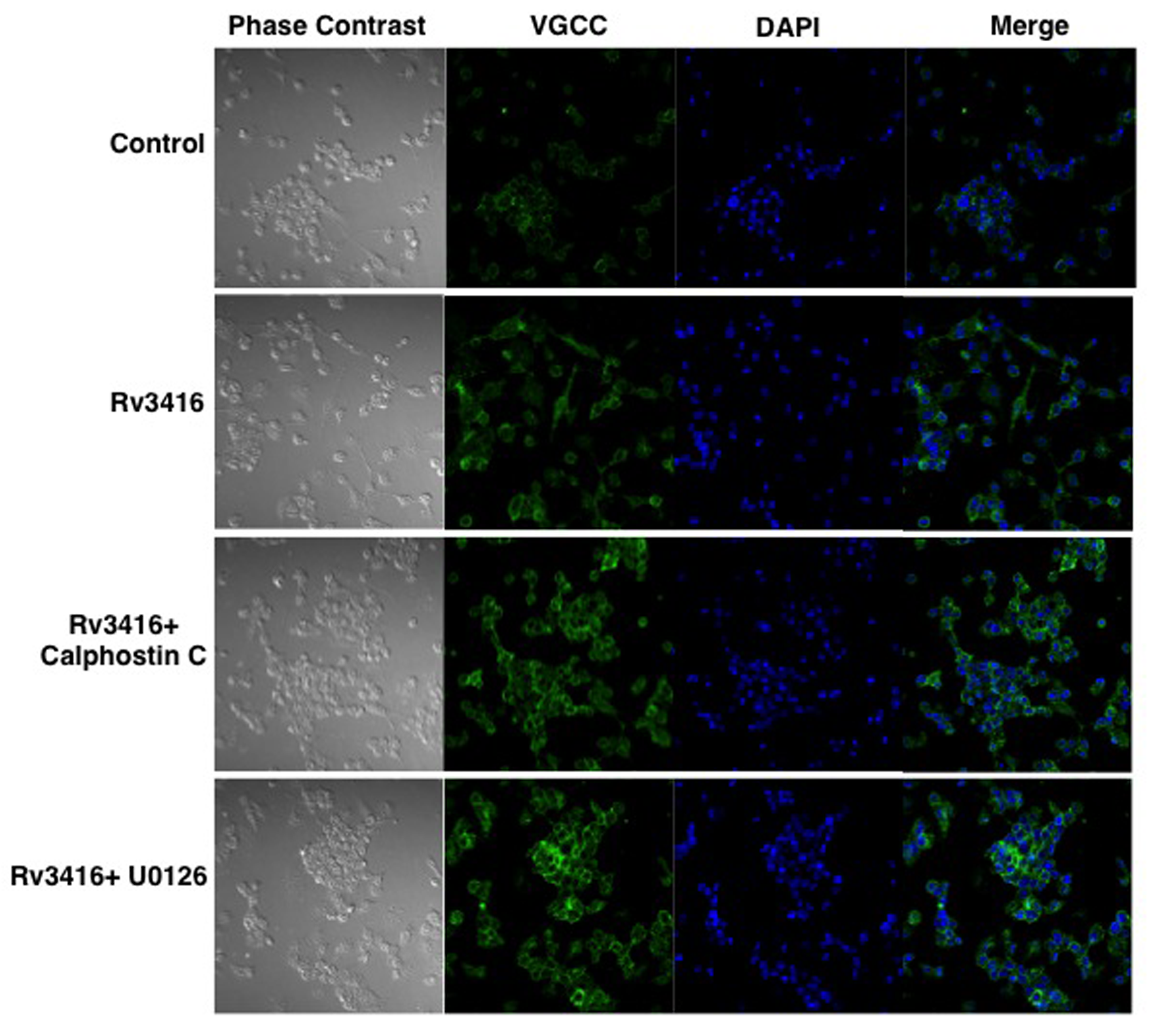

Supplement: Figure S3 — Confocal images of L-type VGCC following stimulations with Rv3416. THP1 cells were stimulated overnight with 50 ng/ml PMA followed by Rv3416 stimulation with or without indicated reagents for 72h. L-type VGCC expression was monitored by confocal imaging as described in Experimental Procedures. A representative image of 10 fields is shown. (TIF) [file pone.0096427.s003.tif]
